# Phytotoxicity of 4,8-Dihydroxy-1-tetralone Isolated from *Carya cathayensis* Sarg. to Various Plant Species

**DOI:** 10.3390/molecules191015452

**Published:** 2014-09-26

**Authors:** Xian-Xian Li, Min-Feng Yu, Xiao Ruan, Yu-Zhu Zhang, Qiang Wang

**Affiliations:** 1 Ningbo Institute of Technology, Zhejiang University, Ningbo 315100, China; 2 Department of Chemical and Biological Engineering, Zhejiang University, Hangzhou 310058, China

**Keywords:** *Carya cathayensis* Sarg., phytotoxicity, 4,8-dihydroxy-1-tetralone, bioassay

## Abstract

The aqueous extract from *Carya cathayensis* Sarg. exocarp was centrifuged, filtered, and separated into 11 elution fractions by X-5 macroporous resin chromatography. A phenolic compound, 4,8-dihydroxy-1-tetralone (4,8-DHT) was isolated from the fractions with the strongest phytotoxicity by bioassy-guided fractionation, and investigated for phytotoxicity on lettuce (*Latuca sativa* L.), radish (*Raphanus sativus* L.), cucumber (*Cucumis sativus* L.), onion (*Allium cepa* L.) and wheat (*Triticum aestivum* L.). The testing results showed that the treatment with 0.6 mM 4,8-DHT could significantly depress the germination vigor of lettuce and wheat, reduce the germination rate of lettuce and cucumber, and also inhibit radicle length, plumule length, and fresh weight of seedlings of lettuce and onion, but could significantly promote plumule length and fresh weight of seedlings of cucumber (*p* < 0.05). For the tested five plants, the 4,8-DHT was the most active to the seed germination and seedling growth of lettuce, indicating that the phytotoxicity of 4,8-DHT had the selectivity of dosage, action target (plant type) and content (seed germination or seedling growth).

## 1. Introduction

The phenomenon of inhibition of one plant’s growth by chemicals released by another plant into environment is generally defined as allelopathy [1]. The term “allelopathy” was first use by Hans Molisch from a physiological perspective to describe the effect of ethylene on fruit ripening [2]. Allelopathic effects are mainly determined by the dose and property of the exuded chemicals, mostly consisting of some secondary metabolites with phytotoxicity, and also by various other environment factors [3,4]. Allelochemicals, delivered through decomposition, volatilization, leaching and root exudation [5], play an important role in the distribution of plant populations [6], the succession of communities, as well as the nutrient chelation [7], and was also suggested as a mechanism driving exotic plant invasion [8]. Found mainly near Tianmu Mountain in the Zhejiang Province of China (30°18'30''–30°24'55''N, 119°23'47''–119°28'27''E) [9], *Carya cathayensis* Sarg. is famous for its daintiness and nutritional content, and has generated increasing interest as a healthy foodstuff to decrease the risk of heart disease [10]. After collecting the fruits of *C. cathayensis*, the exocarp as a forest residue were piped up along the hillside. When the leached exocarp solution flowed down the mountainside, weeds and low shrubs along the flow path and nearside gradually withered and died, suggesting a phytotoxic phenomenon. Phytotoxicity could be a starting point for investigation of allelopathy, and also might be the function of static (*i.e.*, the existing concentration in soils) and dynamic (*i.e.*, the renewal rate) availability of allelochemicals [11,12].

In the last decade, the study of allelopathy has made significant progress. Today, more and more papers on this subject are being published in high impact journals [13]. Recent advances in our efforts to isolate and identify trace amount of biologically active substances including various allelochemicals have encouraged us to study allelopathy deeper and farther, especially in controlled environments [14]. Obviously, it was necessary to collect, extract, separate and then analyze allelochemicals in the natural environment to bioassay their phytotoxicity. Currently, ecologists try to identify and gain pure allelochemicals from complex mixtures by various technologies including UPLC, GC, LC-MS, GC-MS, GC-MS-MS, TLC, CE, paper chromatography, *etc.* [15,16,17]. In order to answer if and how allelopathy influences plant interactions and invades plant, however, multidisciplinary efforts involving plant ecology, genetics, physiology, biochemistry, soil science, microbiology and so on must be made to address this complex research area [18,19].

Over the last years, *Juglandaceae* [9], the constituent of *C. cathayensis* as a member of the walnut family, has been shown to include five flavonoids (cardamonin, pinostrobin chalcone (PC), wogonin, chrysin, pinocembrin) in the leaves, and α-tetralonyl glucosides from the fresh rejuvenated fruit [20]. Previous studies showed that cathayenone A isolated from *C. cathayensis* husk exhibited obvious antifungal activities [21]. However, the chemical composition of *C. cathayensis* exocarp has not been reported so far. The present study will describe the process of isolation of 4,8-dihydroxy-1-tetralone (4,8-DHT) and investigate the phytotoxicity activities of this novel compound, to provide a basis for development and utilization of *C. cathayensis* exocarp and 4,8-DHT as allelochemicals.

## 2. Results and Discussion

### 2.1. Phytotoxicity of C. cathayensis Exocarp

The overall water extract (8.0 ± 0.1 g) of *C. cathayensis* exocarp was absorbed on a macroporous resin column, and 11 fractions eluted with ethanol solutions in ascending order of ethanol proportion were evaporated to afford dry residues weighing 5.3708 ± 0.043 g, 0.8605 ± 0.034 g, 0.3805 ± 0.022 g, 0.3158 ± 0.015 g, 0.1807 ± 0.008 g, 0.1325 ± 0.031 g, 0.1051 ± 0.016 g, 0.0981 ± 0.014 g, 0.0966 ± 0.008 g, 0.0997 ± 0.012 g, 0.1618 ± 0.026 g, respectively. The total eluted residue weighed 7.8031 ± 0.062 g. Each data is the mean of three replicates ± SD.

#### 2.1.1. Effects on Seed Germination of Lettuce

**Figure 1:**
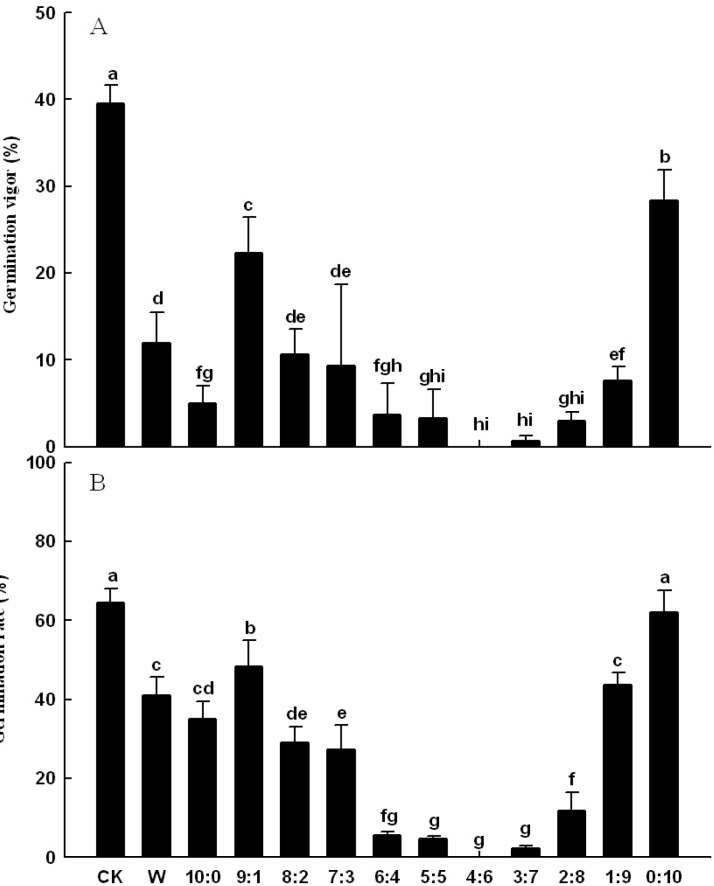
Effects of *C. cathayensis* exocarp extract and elution fractions on lettuce seed germination (**A**) germination vigor, (**B**) germination rate. Data are the mean ± SD values (n = 3). Final means with different letters are significantly different at *p* = 0.05.

Effects of *C. cathayensis* exocarp extract and eluting fractions on lettuce seed germination were measured by germination rate and germination vigor (Figure 1). Using distilled water as a reference (CK), germination vigor was significantly inhibited by the aqueous extract (W) and 11 eluted ethanol solution fractions, and the eluting fraction of 60% ethanol solution completely inhibited seed germination vigor (*p* < 0.05, Figure 1A). Germination rate was also significantly inhibited by the aqueous extract and the 10 eluted fractions, and the fraction eluted by 60% ethanol solution completely inhibited seed germination rate (*p* < 0.05, Figure 1B). The three fractions eluted by the solutions with the water/ethanol volume ratio of 5:5, 4:6 and 3:7, obviously demonstrated stronger inhibition to seed germination of lettuce than the other fractions.

#### 2.1.2. Effects of Extract on Seedling Growth of Lettuce

**Figure 2:**
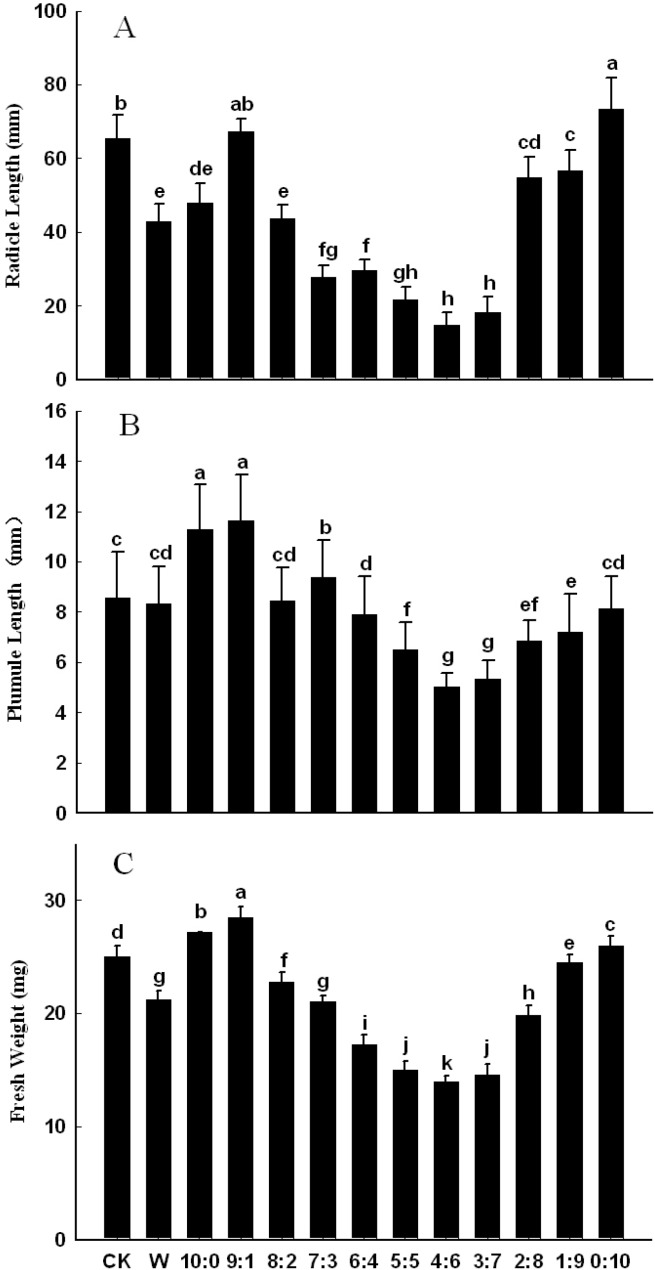
Effects of *C. cathayensis* exocarp extract and elution fractions on lettuce seedling growth: (**A**) radicle length; (**B**) plumule length; (**C**) fresh weight of seedling. Data are the mean ± SD values (n = 3 per group). Final means with different letters are significantly different at *p* = 0.05.

Effects of *C. cathayensis* exocarp extract and eluted fractions on lettuce seedling growth were measured with regard to radicle length, plumule length and fresh weight of seedling (Figure 2). Compared with the control group, the ethanol free water fraction significantly promoted and the fraction elutied by the solution with a water/ethanol ratio of 9:1 slightly promoted the radicle growth, while the other eluted fractions and overall water extract significantly inhibited the radicle growth of lettuce, and the fractions corresponding to the eluents with water/ethanol ratios of 4:6 and 3:7 showed the strongest inhibitory effects (*p* < 0.05, Figure 2A). As shown in Figure 2B, the fractions eluted by the solutions with water/ethanol ratios of 10:0, 9:1 and 7:3 significantly stimulated the plumule growth, but the fractions eluted with water/ethanol ratios of 8:2 and 0:10, as well as the overall water extract, slightly inhibited the plumule growth, and other eluting fractions significantly inhibited the plumule growth of lettuce, for which the inhibitory effects of the fractions corresponding to water/ethanol ratios of 4:6 and 3:7 were the strongest (*p* < 0.05). 

As shown in Figure 2C, the fractions eluted by solutions with water/ethanol ratios of 9:1, 10:0 and 0:10 significantly increased, but other eluting fractions and the overall water extract significantly reduced the fresh weight of seedlings, and the eluting fraction with a water/ethanol ratio of 4:6 exhibited the strongest inhibition (*p* < 0.05). In sum, the fractions eluted by the solutions with water/ethanol ratios of 5:5, 4:6 and 3:7 showed very strong inhibition to seed germination and seedling growth of lettuce, indicating that that there might exist some active phytotoxic chemicals in them.

### 2.2. Isolation and Identification of 4,8-DHT

#### 2.2.1. Extraction and Purification of 4,8-DHT

**Figure 3:**
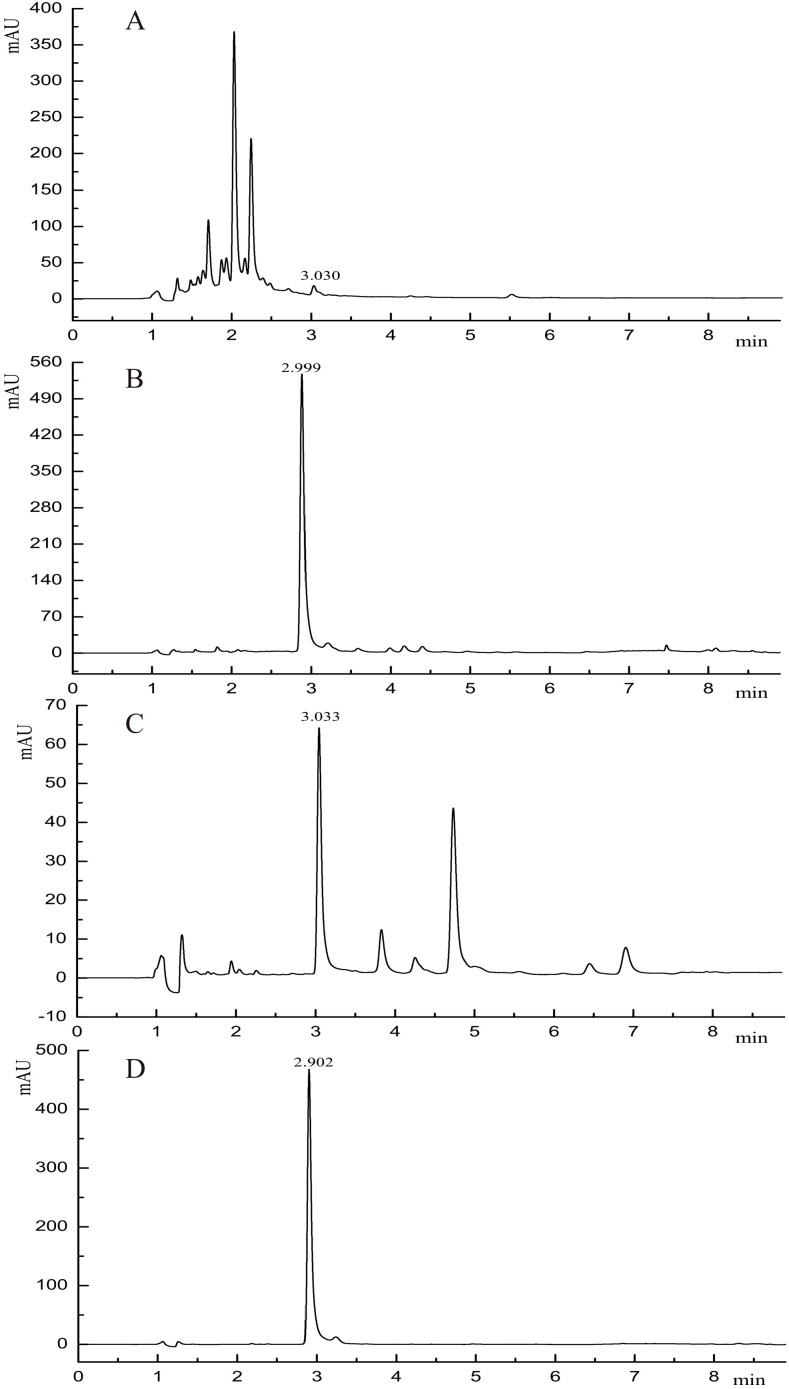
UPLC chromatograms of three eluction fractions and purified sample: (**A**) fraction 5:5; (**B**) fraction 4:6; (**C**) fraction 3:7; (**D**) purified sample.

UPLC analysis of those eluting fractions with the stronger inhibitory activity was conducted. A compound with retention time of ~3 min could be found in the chromatograms of all the three fractions, and its content in the fraction with regard to water/ethanol ratio of 4:6 was 76.15% (Figure 3A–C).

After purification by silica gel and Sephadex LH-20 column chromatography, the residue was recrystallized from EtOAc to give colourless block crystals with a purity of 99.8% (Figure 3D).

#### 2.2.2. Identification and Characterization of 4,8-DHT

The above compound in Figure 3D was identified and characterized by GC-MS, ^1^H-NMR and ^13^C-NMR analysis and comparison with literature data.

**Figure 4:**
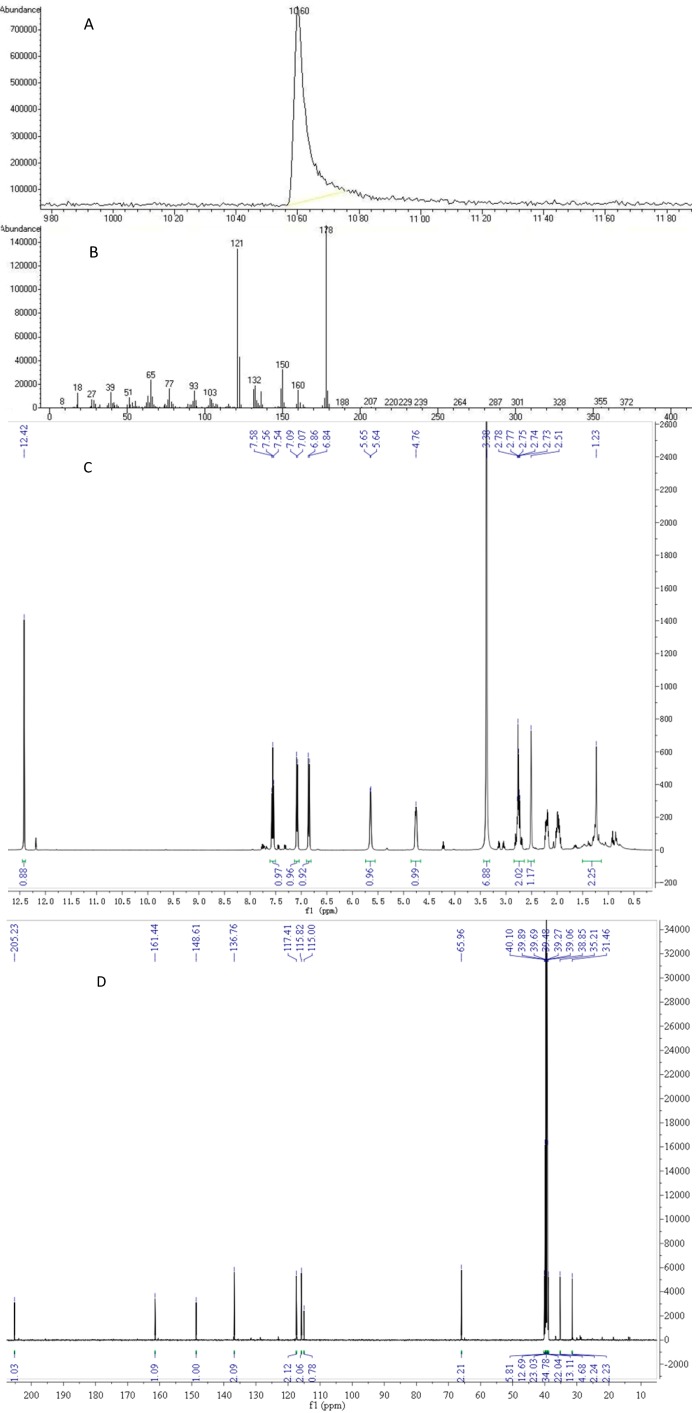
Structure identification of 4,8-DHT: (**A**) and (**B**) GC-MS spectra; (**C**) ^1^H-NMR spectra; (**D**) ^13^C-NMR spectra.

GC-MS analysis determined its molecular weight to be 178 g/mol, and NMR analyses identified its structure from the chemical shifts as follows: ^1^H-NMR: 1.98 (m, 1 H, 3-C*H*_2_), 2.20 (m, 1 H, 3-C*H*_2_), 2.75 (m, 2 H, 2-C*H*_2_), 4.75 (m, 1 H, 4-C*H*), 5.64 (d, *J* = 4.0 Hz, 1 H, 4-O*H*), 6.84 (d, *J* = 8.0 Hz, 1 H, 7-C*H*), 7.08 (d, *J* = 4.0 Hz, 1 H, 5-C*H*), 7.55 (t, *J* = 8.0 Hz, 1 H, 6-C*H*), 12.41 (s, 1 H, 8-O*H*). ^13^C-NMR: 31.50 (3-*C*), 35.25 (2-*C*), 66.01 (4-*C*), 115.04 (8'-*C*), 115.87 (7-*C*), 117.45 (5-*C*), 136.80 (6-*C*), 148.66 (4'-*C*), 161.48 (8-*C*), 205.28 (1-*C*). It was found that these spectral data coincided with the data in literature, which confirmed the structure of 4,8-dihydroxy-1-tetralone (4,8-DHT, Figure 4) [22].

### 2.3. Phytotoxicity of 4,8-DHT

#### 2.3.1. Effects of 4,8-DHT on Seed Germination of Five Plants

**Figure 5:**
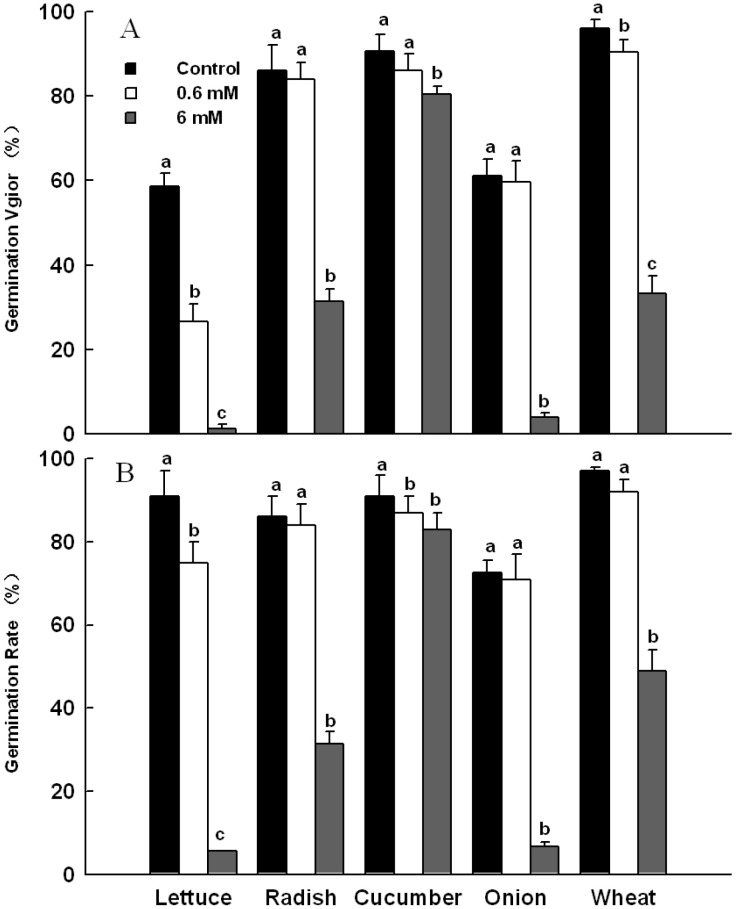
Effects of 4,8-DHT on five plant seed germination: (**A**) germination vigor, (**B**) germination rate. Data are the mean ± SD values (n = 3 per group). Final means with different letters are significantly different at *p* = 0.05.

Effects of 4,8-DHT on seed germination of five plants were measured in terms of germination rate and germination vigor (Figure 5). Compared with the control reference without 4,8-DHT, the solution with 0.6 mM 4,8-DHT significantly reduce germination vigor of lettuce and wheat, but only slightly reduced those of other tested plants (*p* < 0.05); the solution with 6 mM 4,8-DHT significantly reduced germination vigor of all tested plants, where the germination vigor of lettuce was the most sensitive to reduction by high concentration 4,8-DHT solution (Figure 5A). For the effect on germination rate of the plants, the solution with 0.6 mM 4,8-DHT significantly reduced germination rate of lettuce and cucumber but only slightly reduced those of other tested plants; the solution with 6 mM 4,8-DHT significantly inhibited germination rate of all tested plants (Figure 5B). The germination rate of lettuce was the most sensitive to the inhibition by 4,8-DHT treatment and that of cucumber almost did not vary with increasing the 4,8-DHT concentration in the treatment solution.

#### 2.3.2. Effects of 4,8-DHT on Seedling Growth of Five Plants

**Figure 6:**
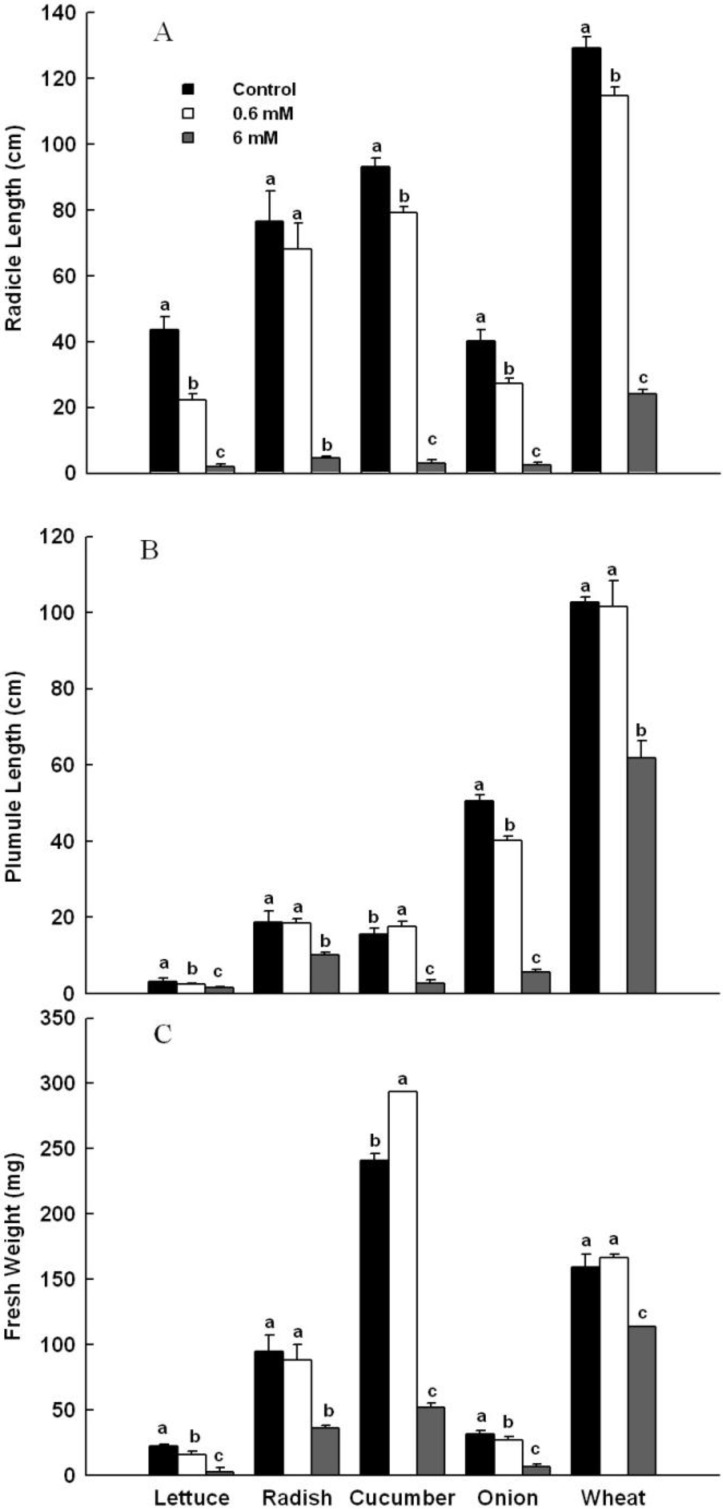
Effects of 4,8-DHT on five plants seedling growth: (**A**) radicle length; (**B**) plumule length; (**C**) fresh weight of seedling. Data are the mean ± SD values (n = 3 per group). Final means with different letters are significantly different at *p* = 0.05.

Effects of 4,8-DHT on seedling growth of five plants were measured in terms of length of radicle and plumule as well as fresh weight of seedlings (Figure 6). Compared with the solution containing no 4,8-DHT, the solution with 0.6 mM 4,8-DHT significantly inhibited radicle length of all tested plants except radish, and the solution with 6 mM 4,8-DHT significantly inhibited radicle length of all test plants (Figure 6A).

As shown in Figure 6B, the solution with 0.6 mM 4,8-DHT significantly promoted plumule length of cucumber, but displayed no significant promotion of that of radish and wheat, and significantly inhibited plumule length of lettuce and onion; the solution with 6 mM 4,8-DHT significantly reduced plumule length of all plants. The data in Figure 6C shows that the solution with 0.6 mM 4,8-DHT caused a significant increase of fresh weight of cucumber seedlings, no significant effect on radish and wheat seedlings, and produce a weight significant decrease of lettuce and onion seedling, while the solution with 6 mM 4,8-DHT significantly decreased fresh weight of seedlings of all plants. Among the tested plants, seedling growth of lettuce was the most sensitive to 4,8-DHT treatment.

4,8-Dihydroxy-1-tetralone (**1**) has a pair of enantiomers found in both fungi and plants, the (–)-**1** enantiomer is commonly named regiolone and the (+)-**1** isomer is called isosclerone. According to *ab initio* calculations of ORs and ECD spectra, the absolute configurations of the two naturally occurring enantiomeric naphthalenones were assigned as (R) for (–)-regiolone and (S) for (+)‑isosclerone [23]. The green husks, leaves, stem and bark of the genus *Juglans* (*Juglandaceae*) have been widely used as folk medicines for the treatment of cancer and dermatosis in Korea, Japan and China since ancient times. The green walnut husks (*Juglans regia* L.), named Qin-Long-Yi in Chinese, is a traditional herbal medicine which has long been used for clearing heat, eliminating toxin, alleviating pain and treating skin disease [24]. Several diarylheptanoids and regiolone isolated from the extracts of the green walnut husks have been studied for their structure and various biological activities such as antitumor, antiinflammatory, antifungal, antibacterial properties. They also inhibit the NF-kB activation, NO, TNF- production, free radical scavenging activities, and related effects [25,26,27]. Phytotoxicity tests and morphological investigations on plant species of horticultural interest indicated that regiolone as principal component of walnut husk washing waters could induce a concentration‑dependent stimulating effect on the growth of radish, lettuce cv. cavolo Napoli up to 165%, and elicit an opposite inhibitory effect up to 70% on spinach and lettuce cv. Gentilina [28]. Isosclerone [(+)-1] was first isolated from *Sclerotinia sclerotium* as a new bioactive metabolite in a plant growth regulating test, later from *Scytalidium* species and as a phytotoxin produced from *Scolecotrichum graminis*, the causal agent of a leaf streak disease in orchard grass [29,30]. Isosclerone was also isolated from other fungal and plant species, it was reported as a phytotoxin of *Botrytis cinerea*, known as a pathogen of a number of crops, especially as the pathogen to the gray mold rot of grapes, as an antitumor metabolite from *Penicillium diversum* var. aurem [31,32]. 4,8-DHT isolated from the exocarps of *C. cathayensis* was confirmed as a racemate (1:1).

In this preliminary investigation, bioassay-guided fractionation of an aqueous extract of *C. cathayensis* exocarps lead to the finding of the key active component 4,8-DHT, proving bioassay to be an efficient method for this purpose. The fraction eluted by the solution of water/ethanol at a volume ratio of 4:6 demonstrated the strongest inhibitory intensity, in comparison with other eluting fractions. UPLC analysis revealed the existence of an active compound which was further identified to be 4,8-DHT by MS-GC and NMR analyses. For the moment, we would not exclude that there might exist other phytotoxic chemicals in water extract of *C. cathayensis* exocarps. However, the eluting fraction with regard to the solution of water/ethanol at ratio of 4:6 displayed a more important research value.

Higher plants release a diversity of allelochemicals into the environment, including phenolics, alkaloids, long chain fatty acids, terpenoids and flavonoids [33]. In this study, we isolated a phenolic substance, 4,8-DHT, from the exocarps of *C. cathayensis* as the major active compound responsible for the observed phytotoxicity. Seed germination and seedling growth studies using phytochemical extracts are most widely used to determine the phytotoxic potential in vegetation [34]. Crop seeds are commonly selected for use in phytotoxic bioassays, because they satisfy a number of selection criteria: they are readily available, affordable, repeatable and reliable; and they germinate quickly, completely, and uniformly. In this study, we selected five crops as test species. Since the content of 4,8-DHT in exocarps of *C. cathayensis* was about 0.25 mg/g, one kilogram of exocarps soaked in ten kilograms of water could generate the solution of 4,8-DHT at 1.4 mM concentration. According to this, we selected a concentration range of 4,8-DHT in treatment solution. The treatment with 0.6 mM 4,8-DHT could significantly reduce germination vigor of lettuce and wheat, germination rate of lettuce and cucumber, and could also significantly inhibit radicle length, plumule length, and fresh weight of seedlings of lettuce and onion. The treatment with 6 mM 4,8-DHT could significantly inhibit the progression of seed germination and seedling growth of the test plants. On the contrary, the treatment with 0.6 mM of 4,8-DHT could significantly promote plumule length and fresh weight of seedlings of cucumber. For seed germination and seedling growth, lettuce was the most sensitive to 4,8-DHT treatment in the five test plants. In summary, the allelopathy must be an interaction between a pair of donor and receptor, and different receptors have variable sensitivity to the same donor; an allelochemical might have the duality of inhibition and promotion to plant growth, and the action intensity and direction could vary with its amount.

Chemicals from a plant alone are not sufficient to ensure their allelopathic potential. Abiotic and biotic environmental conditions determine the allelopathic potential of chemicals in soil [6]. Recent studies have deepened our understanding of allelopathy by examining it in environmental, biogeographic, and evolutionary contexts [35,36,37]. Field examination of intraspecific chemical inhibition of water extract of *C. cathayensis* exocarps and 4, 8-DHT might yield further insights into the role of allelopathic potential of chemicals in the ecosystem. Furthermore, the mechanisms that 4,8-DHT induces growth stress and alters the biochemical and physiological processes needed to be determined. Degradation of 4,8-DHT in the natural environment should be studied to confirm whether 4,8-DHT was completely friendly to environment before it was developed.

## 3. Experimental Section

### 3.1. Plant Materials

Exocarps of *C. cathayensis* were collected from Chun-an country in Zhejiang province (29°22'~29°50'N/118°34'~119°15'E). The selected exocarps were not exposed to rain, otherwise some active compounds soluble in water would be washed away. After some sundries such as nut fragments were picked out, the exocarps were kept in a cool and well-ventilated place. Seeds of lettuce (*Latuca sativa* L.), radish (*Raphanus sativus* L.), cucumber (*Cucumis sativus* L.), onion (*Allium cepa* L.) and wheat (*Triticum aestivum* L.) were purchased from the market in Ningbo of China, and used for bioassay.

### 3.2. Extraction and Isolation of Active Compounds

The ground exocarps of *C. cathayensis* were soaked in distilled water (1 g per 20 mL) at room temperature for 48 h. After exhaustive extraction with stirring, the mixture was sieved through cheesecloth and squeezed to extract as much liquid as possible. The liquid was treated by centrifugation at 4000 rpm for 2 min followed by vacuum filtration through Whatman No. 4 filter paper. Then, the filtrates went through a glass column (60 mm × 610 mm) packed with X-5 macroporous resin. After adsorption saturation, the resin column was sequentially eluted with aqueous solutions containing 0%, 10%, 20%, 30%, 40%, 50%, 60%, 70%, 80%, 90% and 100% ethanol (2.5 L per step). All the eleven eluants were respectively concentrated by decompression into the solutions with concentration about 2.0 mg/mL for testing the allelopathic activity on lettuce. Among them, three fractions with stronger allelopathy activity were analyzed by UPLC. One component with high content was targeted at same retention time, and then was separated through silica gel column with the eluted solution of petroleum ether/ethyl acetate (6:4). Quantitatively, the targeted component of 2.5 ± 0.2 g was obtained from *C. Cathayensis* exocarps of 10 kg. After further purification with Sephadex LH-20 column chromatography, the substance was recrystallized in EtOAc to form the colourless block crystals with ${\left[\text{α}\right]}_{D}^{20}$ = ± 0° (c1.3, CH_2_Cl_2_) and m.p. 366–368 K. For the identification of this active substance, GC-MS analysis showed a molecular ion peak at *m/z* 178 Daltons, and both ^1^H and ^13^C-NMR gave a complete and reliable assignment signals.

### 3.3. Instrumental Analysis

The active compound was identified by GC-MS, ^1^H- and ^13^C-NMR. An Agilent 6890N/5973 gas chromatograph-mass spectrometer equipped with a HP-5MS column (30 m × 0.25 mm I.D × 0.25 μm) was used for the identification of the active compound. Gas chromatography was operated under an initial oven temperature of 80 °C for 2 min, then programmed to 180 °C at a rate of 15 °C /min followed by 5 min at 180 °C, and 25 °C/min to 280 °C for 5 min of final isotherm. The carrier gas helium flowed through the column at a rate of 1.0 mL/min. The splitless injection was adopted with the sample volume of 10 μL. The temperature of injector and detector was maintained at 280 °C. The mass spectrometer was operated at 70 eV with the scan range of m/z ratio from 30 to 550.

Chromatographic separation was carried out on an Agilent 1290 UPLC with a C18 reversed-phase column (ZORBAX Eclipse Plus C18, 2.1 × 150 mm, 1.8 μm) (Agilent, Santa Clara, CA, USA) kept at 30 °C. Detection was performed with a diode array detector set at 254 nm. The mobile phase consisted of: (A) 0.5% acetic and (B) acetonitrile, and the optimum efficiencies of separation were achieved with the elution gradient which, as solvent B proportion, was as follows: 0–5 min, 30%; 5–7 min, 30%–85%; 7–9 min, 85%. The flow was 0.3 mL/min and the injection volume was 1.5 μL.

^1^H-NMR (400 MHz) and ^13^C-NMR spectra (100 MHz) were recorded in NMR spectrometer (Bruker Ac-400 spectrometer). Samples were dissolved in DMSO and chemical shifts were reported in parts per million (ppm) relative to an internal standard of tetramethylsilane.

### 3.4. Bioassay

Stock 4,8-DHT solutions of 100 mM were prepared by dissolving pure 4,8-DHT in distilled water. Using 0 mM 4,8-DHT as control reference for bioassays, two diluted stock solutions with 4,8-DHT content of 6 and 0.6 mM were used as treatment solutions. Experiments of seed germination and seedling growth were conducted according to ISTA (2010) [38].

#### 3.4.1. Effects of 4,8-DHT on Seed Germination

100 grains of surface-sterilized lettuce seeds were placed in each sterile Petri dish (15 cm diameter) lined with Whatman No. 3 filter paper in three replicates. Ten mL of treatment solutions and distilled water as control were added to each Petri dish. The Petri dishes were placed in programmable illuminated incubator with an L/D cycle of 12 h/12 h and a temperature cycle of 25/15 °C. Treatments were carried out in a complete randomized design with three replicates for each treatment. Germination (radicle emergence) was measured 4 and 7 days after treatment. To test the effects of 4,8-DHT on seed germination of lettuce, radish, cucumber, onion and wheat, we conducted experiments similar to those described above. Germination (radicle emergence) was measured in 4 and 7 days after treatment for lettuce, 4 and 10 days for radish, 4 and 8 days for cucumber and wheat, 6 and 12 days for onion.

#### 3.4.2. Effects of 4,8-DHT on Seedling Growth

Pre-germination of lettuce seeds were conducted in plastic boxes (20 × 15 × 10 cm) lined with Whatman No. 3 filter paper for 3–4 days until radicle emergence. One hundred successfully germinated seeds were placed in Petri dishes in three replicates and 10 mL treatment solutions and distilled water as control were added to each Petri dish. Seedlings were incubated in programmable illuminated incubator (incubation conditions were the same as seed germination). Five seeds were randomly taken out from each Petri dish and the length of plumule and radicle were measured with a vernier caliper (GB/T 1214.2-1996, Measuring Instrument LTD, Shanghai, China). Fresh weight of seedlings was also recorded (Mettler Toledo Instrument Ltd., Boston, MA, USA). The measurements were taken on once every two days after incubation and continued for a total of 18 days. Bioassays of 4,8-DHT on seedling growth of five tested species were conducted with the same procedure as above. Plumule, radicle length and fresh weight of seedling were measured once every two days after incubation and continued for a total of 18 days.

### 3.5. Statistical Analysis

We calculated germination rate and germination vigor for each of the five tested species. The percentage of germinated seeds measured in the fourth day for lettuce, radish, cucumber, wheat and the sixth day for onion was counted as germination vigor, while the percentage of germinated seeds measured in the seventh day for lettuce, the eighth day for cucumber and wheat, the tenth day for radish, and the twelfth day for onion was counted as germination rate. The significant differences among treatment solutions and control on seed germination and seedling growth of test species were first examined by ANOVA (*p* < 0.05) and then analyzed using Fisher’s test at *p* < 0.05 level (analyses were performed using the SPSS statistical package v.20, IBM Corp, Armonk, NY, USA).

## 4. Conclusions

The phenolic compound 4,8-dihydroxy-1-tetralone was found to be a key phytotoxic chemical in the exocarp of *C. cathayensis* and demonstrated significant phytotoxicity to seed germination and seedling growth of plants lettuce, radish, cucumber, onion and wheat. The phytotoxic intensity depended on the 4,8-DHT amount and the tested plant. Whereas a high amount of 4,8-DHT (6 mM) generally exhibited an inhibition to seed germination and seedling growth of each plant, small amounts of 4,8-DHT (0.6 mM) actually promoted seedling growth of cucumber, indicating that the phytotoxicity of 4,8-DHT had the dosage, action target (plant type) and content (seed germination or seedling growth) selectivity. It is expected that results of this investigation could establish a basis for the development and utilization of the exocarp of *C cathayensis* and 4,8-DHT.
